# Living With Dementia: Flipping Stigma on Its Ear

**DOI:** 10.1093/geroni/igaa057.135

**Published:** 2020-12-16

**Authors:** Deborah O'Connor, Alison Phinney, Jim Mann, Habib Chaudhury, Kishore Seetharaman, Ania Landy, Malcolm Paulina

**Affiliations:** 1 University of British Columbia, Vancouver, British Columbia, Canada; 2 University of Victoria, Surrey, British Columbia, Canada; 3 Simon Fraser University, Vancouver, British Columbia, Canada; 4 University of British Columbia, Vancouver, United States; 5 University of British Columbia, Vancouver, Canada

## Abstract

The language of social citizenship has emerged in the academic literature as one way of shifting the discourse to counter persistent problems of stigma and social exclusion for people with dementia. What this means and how it is experienced however from the perspective of those with dementia remains unclear. As part of a larger Participatory Action Research (PAR) study, an Action group of people with dementia began meeting in June 2019. The group now consists of ten members and meets monthly. The first task of the Action group was to assist in developing a more refined and practical understanding of the construct of social citizenship. Facilitated discussions were guided by the following questions: What are experiences of social citizenship by people with dementia? What kinds of practices and relationships promote the capacity of people with dementia to experience themselves as social citizens? Emerging findings indicate that the stigma is readily identified as a dominant aspect of the experience of living with dementia which needs to be ‘flipped on its ears’. Strategies for countering stigma include recognizing how language can both facilitate and block change, acknowledging dementia as a time of both loss and significant growth, remaining visible as a whole person – equal and also different - and maintaining active participation in one’s own life. These themes tie directly to the components identified in the academic literature of citizenship. However, members of the Action group were clear that the language of social citizenship is neither empowering nor strategic.

